# Treatment by Mechanical Vibration

**Published:** 1903-02-21

**Authors:** 


### Treatment by Mechanical Vibration.

It is perhaps—indeed some would say that it is without a doubt—the duty of the physician to try every method of treatment that is brought forward by responsible people and supported by even a moderate amount of respectable evidence, and the reports which are published from time to time regarding the efficiency of an old method which has lately come somewhat into vogue again, namely, what is called "mechanical vibratory stimulation," may tempt some to experiment with it. At the same time it must be remembered that vibration is a method which has cropped up again and again in years gone by, generally only to be rather shunned by the leaders of the profession, and that, even now in its renaissance, the claims made by those who practise it are somewhat of that all-embracing nature which leads sober-minded people to shake their heads. Let us take the latest essay on the subject. Dr. Maurice F. Pilgrim,* who is in the full swing of what is going on in New York in regard to psychiatry and electro-therapeutics, speaks of this mode of treatment as "the newest and one of the most valuable methods in advanced therapeutics." Mechanical stimulation and vibration have for their aim "the accomplishment of one primary condition, viz., stimulation of the nerve or nerve centres concerned in and controlling the diseased organ, which are found principally in the spinal and sympathetic systems," and he goes on to say that "this is attempted for the double purpose of (1) stimulating and equalising blood currents; and (2) stimulating secretion and excretion, and the lymphatics." Clearly, if it will do these things we have at our disposal a mighty force, so that in the words of Dr. Pilgrim it may be said that "its range of application is almost coextensive with the needs of the ailing physical organism, and is, in varying degree, applicable to almost every form of disease." Such a claim as this is sufficient to make some of us very suspicious, and inclined to tread warily, especially since we are told that the list of diseases in which vibratory treatment has been attended with "results more prompt and satisfactory than by any other recognised methods of treatment," includes the various forms of nervous disorders—neurasthenia, melancholia, insomnia, hysteria, and the like—"in all of which beneficial effects were realised from the first treatments"—a thing quite in accordance with what is often seen in the treatment of such neuroses by other bizarre methods. We are told, moreover, that in scoliosis—which is another great field for therapeutic delusions—"quick results have followed the treatment in every instance, and without employing suspension, casts, braces, or other auxiliary methods." Then we come to the various "congestive forms of pelvic diseases (exclusive of pus sacs)" in which we are told that vibratory treatment has given results "that may well inspire the hope that a brighter day is soon to dawn in the management of this large and troublesome class of cases." Again "Mechanical vibration has been found *uniformly* successful in the treatment of varicose conditions." "Our records show that not a single case of neuralgia, such as myalgia, sciatica, pleurodynia, etc., has failed of speedy and lasting cure under this treatment where it has been persisted in for a reasonable length of time." Even cases of pulmonary tuberculosis in the first stage have, in every case thus far treated, been markedly benefited without employing any other remedial agent whatsoever. "One case in which there was depression below the right clavicle, marked dulness on percussion, with râles and considerable cough and expectoration, showed much improvement after the second treatment." After all this we welcome the modesty which leads Dr. Pilgrim to declare that "too much must not, of course, be claimed for mechanical vibratory treatment." That there is a good deal to be said in favour of "stimulation" by means of mechanical vibration we quite believe. The immediate physical result is sufficiently striking to give much ground for hope that at least some permanent change in blood supply, and perhaps in metabolism, may thereby be set up. We are quite clear, however, that the worst thing that could happen to such a form of treatment would be the formulation of extravagant claims by its professors. That it deserves full and scientific investigation goes without saying. It is, then, very desirable that a careful endeavour should be made to ascertain with some degree of exactitude what are the limits of its powers and utility, and all this should be done under full professional sanction, and with all those guarantees which are given by our great hospitals. Otherwise a form of treatment which, according to Dr. Pilgrim and others, is one of considerable utility, may drift, as so many useful things have done, into the hands of the great unqualified, thereby getting soiled with a taint of quackery which it may be very difficult afterwards to get rid of.

* New York Medical News, Jan. 24.

